# Bone mineral density after implantation of a femoral neck hip prosthesis – a prospective 5 year follow-up

**DOI:** 10.1186/s12891-015-0624-0

**Published:** 2015-08-12

**Authors:** Wolfram Steens, Friedrich Boettner, Rainer Bader, Ralf Skripitz, Alberto Schneeberger

**Affiliations:** Department of Orthopedics, University Medicine Rostock, 18057 Rostock, Germany; Hospital for Special Surgery, New York, NY USA; Endoclinic Zurich and University of Zurich, Zurich, Switzerland

**Keywords:** Femoral neck prosthesis, Periprosthetic bone density, DEXA scan

## Abstract

**Background:**

Bone resorption in the proximal femur due to stress shielding has been observed in a number of conventional cementless implants used in total hip arthroplasty. Short femoral-neck implants are claiming less interference with the biomechanics of the proximal femur. The goal of this study was to prospectively investigate the in vivo changes of bone-mineral density as a parameter of bone remodeling around a short, femoral neck prosthesis over the first 5 years following implantation. The secondary goal was to report on its clinical outcome.

**Methods:**

We are reporting on the changes of bone mineral density of the proximal femur and the clinical outcome up to five years after implantation of a short femoral neck prosthesis. Bone mineral density was determined using dual energy x-ray absorptiometry, performed 10 days, three, 12 and 60 months after surgery. 20 patients with a mean age of 47 years (range 17 to 65) were clinically assessed using the Harris Hip Score. The WOMAC was used as a patient-relevant outcome-measure.

**Results:**

In contrast to conventional implants DEXA-scans overall revealed a slight increase of bone mineral density in the proximal femur in the 12 months following the implantation. The Harris Hip Score improved from an average preoperative score of 46 to a postoperative score at 12 months of 91 points and 95 points at 60 months, the global WOMAC index from 5.3 preoperatively to 0.8 at 12 months and 0.6 at 60 months postoperatively.

**Conclusion:**

At 60 months after implantation of a short femoral neck prosthesis, all regions except one (region of interest #5) showed no significant changes in BMD compared to baseline measurements at 10 days which is less to the changes in bone mineral density seen in conventional implants.

## Background

Mechanical load forces in total hip arthroplasty continuously expose bone to remodeling processes according to Wolff’s law depending on implant size, geometry and stiffness. Muscle activity also has a significant influence on the entire bone density triggering an increase of bone mass and bone strength [1–3]. Conversely, immobilization and inactivity atrophy causes a decrease of bone mass. The degree of bone mineralization is a fundamental determinant of bone quality and correlates with bone stability [4, 5]. But influence of bone mineral density on implant migration after surgery still remains unclear [6]. The Norwegian Arthroplasty Register reports a potential risk of aseptic loosening concomitant to decrease of bone mineral density [7]. Considerable periprosthetic bone resorption in the proximal femur due to stress shielding has been observed after total hip arthroplasty with medullary fixation [8–10]. Therefore, proximal load transfer to the trochanteric region should probably be aimed in modern implant designs. Conventional implants have shown a constant decrease of periprosthetic bone mineral densitiy (BMD) in the proximal femur, as demonstrated by dual-energy x-ray absorptiometry (DEXA) especially over the course of the first year following surgery [11, 12]. Short femoral-neck implants are claiming less interference with the biomechanics of the proximal femur. The mean age of patients requiring total hip replacement is constantly decreasing. The Swedish Hip Arthroplasty Register reports an implant survival of 75 % at 14 years for male patients younger than 50 years, compared to a survival of 84 % for male patients between the age of 60 and 75 years [13]. Although the reasons for failure in the group of young patients is multifactorial, short stemmed femoral shaft prostheses have the theoretical advantage to preserve bone at the initial implantation and ideally maintain this amount of bone over time for upcoming revisions. While long-term results for this group of implants have not been reported, the concept has been supported by in vitro studies using composite and cadaver femora models [14–16]. The primary goal of this study was to prospectively investigate the in vivo changes of bone-mineral density as a parameter of bone remodeling around a short, femoral neck prosthesis over the first 5 years following implantation. The secondary goal was to report on its clinical outcome.

## Methods

**Fig. 1:**
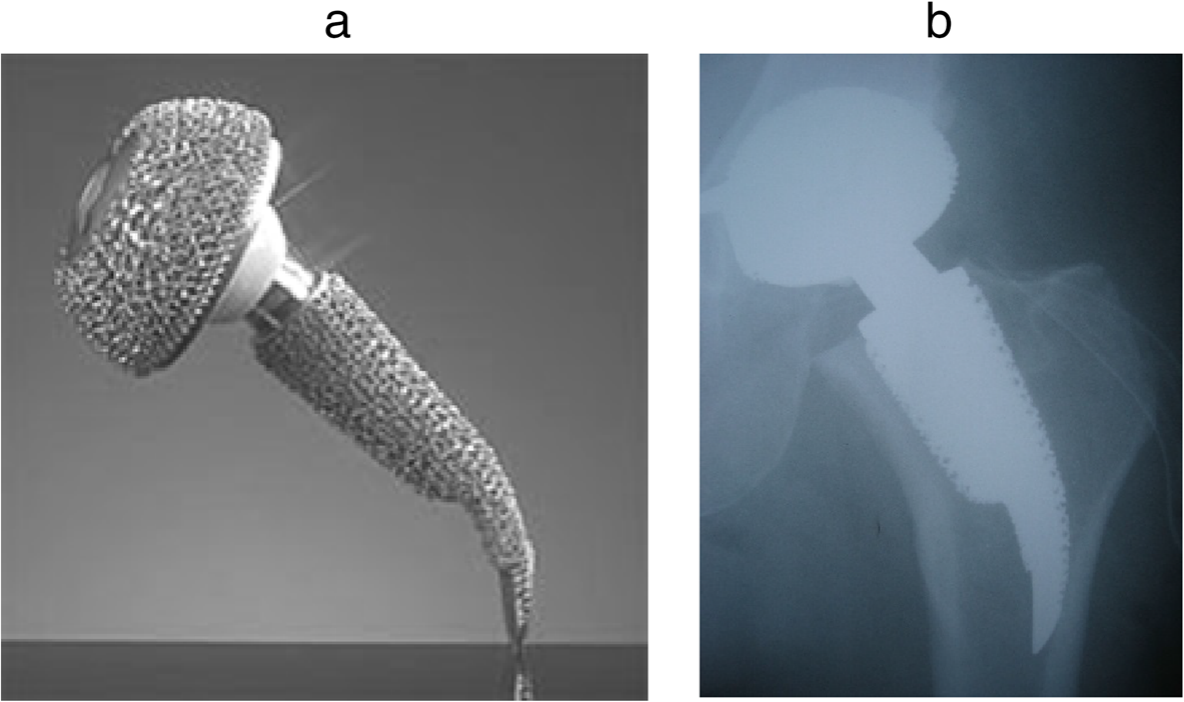
**a, b** CUT femoral neck prosthesis. The short femoral neck implant used in the present study

**Fig. 2:**
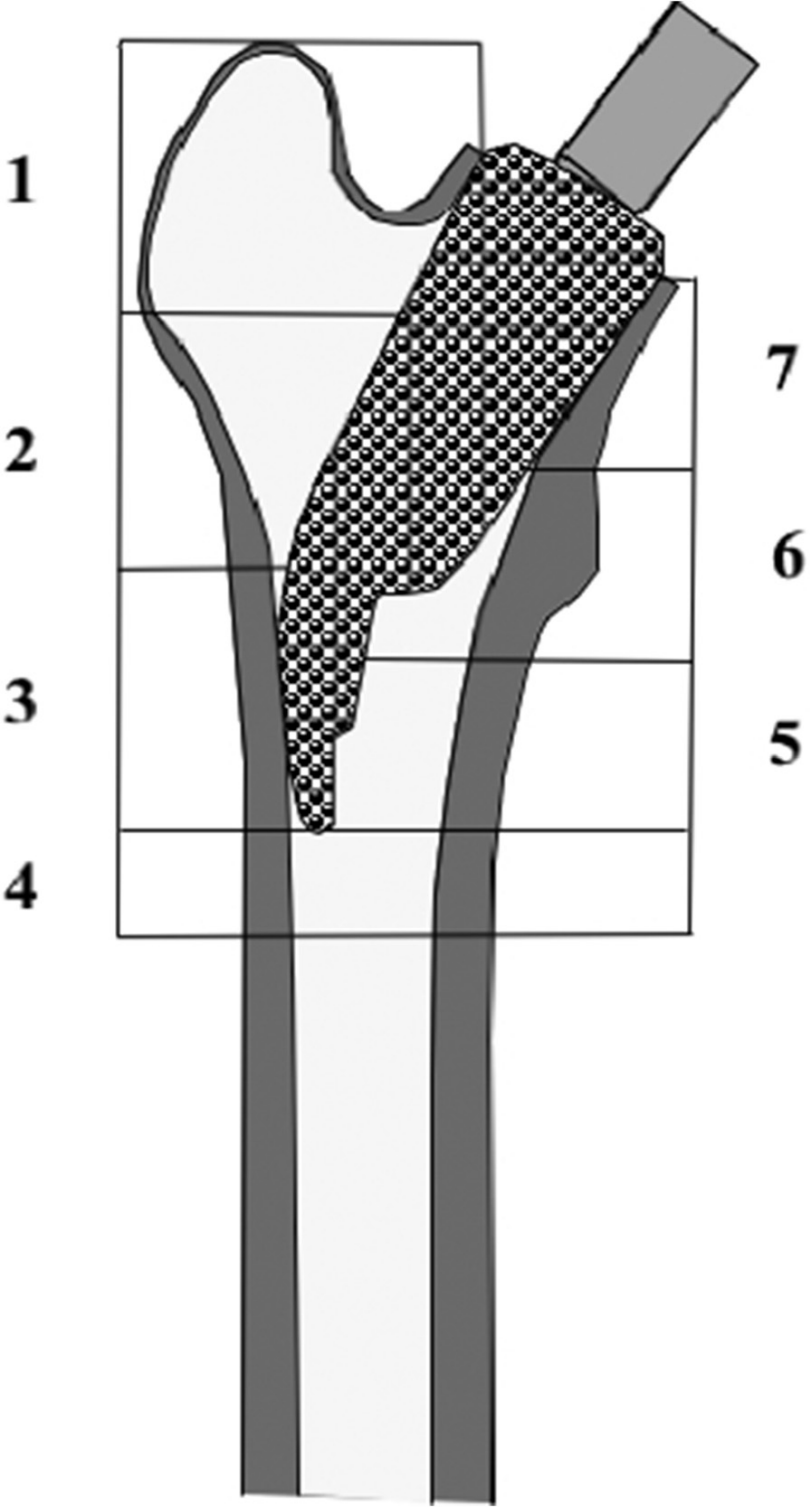
ROI 1–7 (regions of interest). Combination of ROI 1–3 to one medial value as ROImed. Analogue combination of ROI 5–7 to ROIlat. Bone mineral density around the whole implant was calculated as ROIall

20 consecutively treated young aged patients with a cementless short stemmed total hip replacement were included in this study. Indication for primary arthroplasty was osteoarthritis of the hip joint due to developmental dysplasia of the hip in 9 cases, primary oestoarthritis in 7 cases and avascular necrosis of the femoral head in 3 cases. There were 8 female and 12 male patients. The median age at the time of surgery was 49 years (17–67) and at the time of last follow-up examination 54 years (21–73). The average weight and height of the female (and male) patients at the time of surgery were 70 ± 9.9 kg (85 ± 11.8 kg), 169 ± 3.9 cm (177 ± 9.5 cm), Body Mass Index (BMI) 25 ± 3.9 (27 ± 2.8). The right hip was affected in 12, the left in 8 cases. The femoral implant used in all cases was an uncemented, "stemless" ESKA CUT 2000 femoral neck prosthesis (ESKA Orthodynamics, Luebeck, Germany). It is made of cobalt-chrome-molybdenum alloy (CoCrMo) and has a macroporous surface structure (Fig. 1). It’s available in different lengths (50–100 mm) and diameters (19 – 29 mm). The proximal part of the prosthesis is oval in shape. The distal, curved part becomes narrower at its tip. The shape of the prosthesis is neutral for use on both the left and the right side. This implant was used only for patients not older than 65 years and only for those patients that had a physiological collum-center-diaphysis angle (CCD). For implantation of this "stemless" prosthesis, only the femoral head is resected while the complete femoral neck is preserved to support the implant. Its distal part is meant to firm up on the lateral cortical bone just below the greater trochanter. A cold- sealed modular conus adapter (12/14 mm) with adaptable angles (10° and 20°) was used to restore offset and anteversion of the femoral neck. In all cases, a ceramic head was used in combination with a polyethylene-insert (PE) in a cementless press-fit acetabular component. Surgery incidentally was performed by 3 senior consultants of the same department. The post-operative treatment regime included weight bearing as tolerated during a 10 to 14-day inpatient stay and a following three-week stay in a rehabilitation facility. At 3 months follow-up, all patients had been able to bear full weight for at least 6 weeks. Using crutches was mandatory for the first 3 weeks. After institutional review board (Ethikkommission of Westfalian Wilhelms-University of Muenster – No 2008008fS) approval and informed consent, 20 patients were examined by an unbiased examinator preoperatively, at 10 days, 3 months, 12 months and 60 months postoperatively. One patient died after the 12 months-follow-up examination of unrelated causes to surgery. Therefore, only 19 patients were available at last follow-up at 60 months after surgery. The Western Ontario and Mc Master Universities Arthritis Index (WOMAC) and the Harris Hip Score (HHS) as disease specific tests after of total hip replacement were recorded [17, 18]. For this study, the WOMAC's German version was used, which has been shown to be a valid and liable instrument to assess symptoms and physical function disability in patients with hip osteoarthritis [19]. To determine femoral periprosthetic bone density, DEXA was performed at the 10 day-, 3 month, 12 month and 60 month-follow-up, using a Norland Eclipse Scanner (Norland, Ft. Atkinson, WI, USA). Measurements of a calibration phantom were performed daily before scanning the patients. A standardized blinded scanning procedure and positioning of the patients and the replaced hip joint were performed to guarantee a high accuracy of the measurements, as requested by Cohen and Rushton [20]. Densitometric measurement of the non-operated side was performed with each measurement of the operated hip to rule out a possible systemic bone density loss in every case. There were no patients with bilateral total hip replacement. A software designed for the measurement of bone mineral density adjacent to metal implants was used (Norland DxA Version 3.9.4) on a Norland PC (NPC-200). Seven regions of interest (ROI) were determined according to a modified classification of Gruen to respect the specific dimensions of the femoral implant [21] (Fig. 2). Because of the relatively small dimensions of the implant, and the congruously small zones compared to conventional prosthesis, the lateral zones 1, 2 and 3 were later combined to a single lateral value (ROI lat) and the zones 5,6 and 7 combined to a single medial value (ROI med). Bone mineral density around the whole implant was also calculated (ROI all). Periprosthetic BMD was measured longitudinally at the four postoperative follow-ups. At each measurement, the change in BMD was compared with the baseline 10 days after surgery and calculated as the BMD change in percent in each of the 7 primary and 3 combined regions of interest. For statistical analysis JMP IN statistical software (SAS Institute Inc, NC, USA) for Macintosh was used in its version 5.1.2 by a statistician blinded to the study At first the presuppositions for a normal distribution were tested. The Wilcoxon signed-ranks test was used to statistically compare the density changes over the 60 months following surgery. The level of significance for all statistical analyses was set at alpha = 5 %.

## Results and discussion

Bone mineral density of the different regions and percentage of changes are shown in Table 1. Within the first three months after surgery bone mineral density overall showed a slight decrease. A high decrease of about 3 % was recorded in the proximal regions 1 and 6, while the smallest decrease was observed in ROI 3, where the lateral flange of the implant pushes against the lateral cortex. The changes in all regions were statistically significant at 3 months. However, 12 months postoperatively the BMD in all regions had almost normalized close to the initial values recorded 10 days after the index procedure, with the highest increase in ROI 3 (mean +2,8 %, SD: 1.9). In contrast to the medial side, all regions on the lateral proximal femur showed a significant change (ROI 1–3, ROIlat). 60 months after surgery the BMD of all regions on the lateral proximal femur showed a slight, non-significant decrease compared to the values recorded after 12 months respectively 10 days after surgery. Comparing analog values of the medial side, ROI 5 showed only a significant change in bone mineral density 60 months after surgery. Subscores and Global indices of the Harris hip score and the WOMAC are listed in Tables 2 and 3. Significance was measured using the two-sided Student’s *t*-test for paired samples for the WOMAC. This test shows a significant reduction of mean pain, stiffness and function between the pre- and 12 month post-operative scores, and a non-significant change in scores at 60 months. There were no radiographic signs of loosening or migration of the components at radiological follow- up examination three months, one and five years postoperatively in any of the stems and sockets. Evaluation of the sockets on ap radiographs of the pelvis was performed according to DeLee/Charnley [22] and evaluation of the CUT stem according to Engh was performed on ap radiographs as well and an additional lateral view of the proximal femur [23]. The mean preoperative Harris hip score of 45 points showed a distinct increase to 91 points 12 months after surgery and furthermore increased to 95 points after 60 months. The preoperative WOMAC score of 5.3 improved to 0.8 12 months after surgery and showed a further improvement to 0.6 after 60 months. Subscores and Global indices of the Harris hip score and the WOMAC are listed in Tables 2 and 3.

**Table 1:** Bone density and changes between measurements

ROI	10 days		3 mo		10d – 3 mo		12 mo		10d – 12 mo		60 mo		10d – 60 mo	
	mean bone densitiy in	SD	mean bone density in	SD	mean change in %	*p*	mean bone density in	SD	mean change in %	*p*	mean bone density in	SD	mean change in %	*p*
	g/cm2		g/cm2				g/cm2				g/cm2			
1	0.76	0.14	0.73	0.13	−3.35	s	0.75	0.13	−0.76	s	0.74	0.18	−2.51	ns
2	0.81	0.14	0.79	0.14	−2.99	s	0.82	0.14	1.60	s	0.79	0.19	−1.26	ns
3	1.06	0.19.	1.05	0.19	−1.09	s	1.08	0.19	2.84	s	1.05	0.21	−0.009	ns
4	1.60	0.23	1.57	0.22	−2.28	s	1.60	0.23	−0.35	ns	1.57	0.24	−1.32	ns
5	1.52	0.19	1.49	0.19	−3.01	s	1.51	0.19	−0.77	ns	1.43	0.18	−5.43	s
6	1.51	0.20	1.47	0.20	−3.71	s	1.50	0.19	−0.69	s	1.58	0.24	5.13	ns
7	1.08	0.12	1.06	0.12	−2.77	s	1.08	0.12	0.67	ns	1.13	0.19	4.67	ns
1-3 lat	0.82	0.14	0.80	0.14	−2.36	s	0.83	0.14	1.37	s	0.86	0.18	5.00	ns
5-7 med	1.37	0.15	1.10	0.13	−3.18	s	1.13	0.13	−0.40	ns	1.38	0.16	0.73	ns
1-7 all	1.05	0.14	1.02	0.13	−2.74	s	1.05	0.13	0.19	ns	1.18	0.16	1.12	ns

Mean values of bone mineral density and mean values of changes in percent between the 10-day- and 3-month-examinations, as well as between the 10-day- and 12-month-examinations and between the 10-day-and 60-month examinations. *ROI* Regions of Interest 1–7 and combined zones laterally (1–3 lat), medially (5–7 med) and overall (1–7 all). *SD* standard deviation, *s* significant, *ns* not significant (Wilcoxon signed-ranks test, t = 0.05)

**Table 2:** Harris Hip Score

	HHS preop		HHS 12 months		HHS 60 months	
		SD		SD		SD
pain	11.1	7.4	39.6	8.8	41.1	4.4
function	26.9	6.3	43.1	8.6	45.5	3.8
deformity	3.7	0.5	4.0	0.2	4.7	0.0
motion	4.0	0.6	4.9	0.1	4.0	0.1
HHS global	45.6	11.7	88.5	15.1	95.3	22.7

Harris Hip Score preoperatively, 12 and 60 months after implantation of the stem; all values mean; *SD* standard deviation

**Table 3:** WOMAC Score

	WOMAC preop		WOMAC 12 months		WOMAC 60 months	
		SD		SD		SD
pain	5.2	1.1	0.8	1.2	0.5	0.7
stiffness	4.9	1.4	0.9	1.3	0.6	0.7
function	5.7	1.1	0.9	1.1	0.7	0.8
global index	5.3	1.0	0.8	1.2	0.6	0.5

WOMAC preoperatively, 12 and 60 months after implantation of the stem; all values mean; *SD* standard deviation

Modular stems with interchangeable necks have the potential to optimize hip biomechanical parameters. But there is increasing concern regarding the occurrence of adverse local tissue reactions from mechanically assisted crevice corrosion at the neck–stem taper junction [24]. Furthermore mechanical failures such as fractures of the modular neck [25] and dissociation of modular components [26] have been reported in the past. Problematic dual taper stem total hip arthroplasty has been reported to base on various intrinsic and extrinsic causes. A systematic treatment approach according to a risk stratification algorithm previously described should be followed to optimize management of such cases [27].

Although at long-term follow-up conventional femoral stems perform exceedingly well in primary hip arthroplasty, limitations persist, including proximal-distal mismatch, non-ideal load transfer and loss of bone. Investigations in tapered stems have proven a progressive loss of proximal bone density as well in cortical and cancellous parts [28]. The continuing evolution in implant design has led to changes in morphology, materials, surface finishing and tribologic coupling. In addition, increased use of total hip replacement in younger patients prompted to more conservative surgical options in order to preserve as much bone stock as possible. Use of short-stemmed prostheses has considerably increased which can be demonstrated by the development of numerous models of this type of prosthesis from different manufacturers. Short-stemmed femoral implants were mainly designed to achieve proximal load transfer to avoid distal osseointegration, which leads to proximal stress-shielding in conventional implant designs [29–31]. There still is controversy about an exclusive metaphyseal strain distribution in short-stemmed femoral prostheses. While some authors like Fokter published results of proximal load transfer leading to an increasing or persisting bone mineral density after implantation of such implants others like Jahnke and Nysted could not observe this findings but measured BMD atrophy in ROI 1 and 7 at 12 months follow-up [32–38]. The former suggested bionic stem related larger intraoperative greater trochanter resection to be responsible for increasing BMD. DEXA scans are widely accepted to investigate osseointegration after total hip arthroplasty (THA) using different stem designs. Evaluation of bone remodeling in conventional implants according to Gruen can easily be adapted to the evaluation in short stem designs. Factors like gender, age and body weight were found to have certain influence on BMD but there seems to be a consensus of the fact that stem design and mode of fixation remain the major factors [39, 40]. To obtain comparable base line values we used the first postoperative measurement taken 10 days after surgery to avoid possible bias. Furthermore, the methods were standardized and the rotation of the leg was controlled as suggested by studies on the measurement precision of periprosthetic BMD [41]. Previous studies revealed a maximum bone remodeling 6 months after implantation followed by a plateau approximately 12 months after surgery. Further adaptions slowly occur within the following 12–24 months. Thus, the duration of follow-up in our cases should be long enough to reflect any specific changes to the surrounding bone for mid-term studies. No signs of loosening were detected in the radiological analysis. No revision arthroplasty had been performed so far.

Koebke in 2000 [42] published results of strain measurements of the CUT prosthesis in a cadaver model close to physiological behaviors. Steinhauser recorded values of 3 different types of short femoral neck prostheses in composite femora compared to a conventional implant using photoelastic coating techniques. All of the 3 femoral neck prostheses showed less changes in hoop-strains compared to conventional implants. The CUT prosthesis exclusively had significant changes at the tip of the stem only [14]. This corresponds to the data from the present study representing a slight but significant increase of bone mineral density after 12 months. In addition, decreasing hoop-strains were recorded in the lesser trochanter region (ROI 6) which corresponds to decreasing bone mineral density seen in our study after 12 months.

Munting and coauthors showed similar results in an in vitro study with an experimental stubby stem [16]. In a following in vivo study they could show results of bone mineral density in accordance to our study that leveled off after 6 years [43]. In a comparative in vitro trial the straight and the "anatomic" stem both led to a decrease of the longitudinal strains in the proximal femur, while the femoral neck implant mainly led to an increase of measured strains on the lateral side of the greater trochanter. The observed medial strains were closer to physiological values in the "stemless" prosthesis than those of the two full-stem prosthesis [15]. Yamaguchi compared a fully porous-coated stem to a only proximally porous-coated stem. Periprosthetic bone-mineral density was measured with dual-energy x-ray absorptiometry at specific intervals after the operation [44]. In both groups, all ROI had a greater loss of bone-mineral density, compared to the values of our study. Similar results were reported by Nishii and Kröger analyzing different types of conventional cementless implants over the first year after impantation [45, 46]. They all had in common a distinct loss of bone mineral density in ROI 1 and 7 compared to the CUT. Aldinger reported a 25 % decrease of bone mineral density in a conventional cementless Spotorno stem after 5 years which mainly focused on proximal regions ROI1 and 7 [47]. Similar results were published by Brodner with encouraging increase of bone mineral density in ROI 2 to 5 but similar loss in ROI 1 and 7 [12]. Furthermore, some authors reported on custom made or anatomical implants neither having a positive impact on proximal bone stock alterations but showing increased loss of bone mineral density in all Gruen zones [9, 48]. In contrast to other commonly employed models the present study suggests the hypothesis that the CUT prosthesis leads to less proximal bone resorption within the first 5 years after implantation compared to conventional implants. Significant changes of bone mineral density in our study mostly occurred within the first year after surgery. According to previous studies on CUT prostheses clinical results in this study were excellent which might be related to the younger age of our population. Stukenborg-Colsman in a short follow-up study described an improvement of the Harris Hip score from 42.9 points preoperatively to 82.9 points after 1 year [49]. Ender and co-workers as well as Rudert and co-workers showed significantly incresasing HHS results after mid-term follow-up with 93 points after 4.9 years and 95 points after 5 years respectively [32, 50]. The significant improvement of WOMAC from 5.3 to 0.8 points reflects the very good clinical results as well.

## Conclusion

The current study shows that implantation of a short femoral neck stem particularly leads to a significant decreasing bone density in only one region of interest (#5) which is different to the changes in bone mineral density seen in conventional implants. Long-term studies are necessary to analyze the long-term influence of this observation and to identify possible advantages on the revision rate of this type of stem design. The secondary goal of the study reveals an excellent clinical outcome which might be influenced by the younger age of the population and is equal to clinical results of long term established conventional implants. Summing it up beside a good clinical mid-term behavior this implant presents a biomechanic rationale and a perspective of revision able to place it in the foreground of conventional implants especially in younger aged patients. The conclusion of this study is limited because of the relatively small number of non- randomized patient selection and the fact that it is not a clinical trial.

## Abbreviations

BMD: bone mineral density
DEXA: dual-energy x-ray absorptiometry
BMI: body mass index
CoCrMo: cobalt-chrome-molybdenum
CCD: collum-center-diaphysis
PE: polyethylene
WOMAC: Western Ontario and Mc Master Universities Arthritis Index
HHS: Harris Hip Score
ROI: region of interest
THA: total hip arthroplasty
